# Idiosyncratic Hepatocellular Drug-Induced Liver Injury by Flucloxacillin with Evidence Based on Roussel Uclaf Causality Assessment Method and HLA B*57:01 Genotype: From Metabolic CYP 3A4/3A7 to Immune Mechanisms

**DOI:** 10.3390/biomedicines12102208

**Published:** 2024-09-27

**Authors:** Rolf Teschke

**Affiliations:** 1 Department of Internal Medicine II, Division of Gastroenterology and Hepatology, Klinikum Hanau, 63450 Hanau, Germany; rolf.teschke@gmx.de; Tel.: +49-6181-21859; Fax: +49-6181-2964211; 2 Academic Teaching Hospital of the Medical Faculty, Goethe University Frankfurt am Main, 60590 Frankfurt am Main, Germany

**Keywords:** Roussel Uclaf Causality Assessment Method, RUCAM, updated RUCAM, DILI, idiosyncratic drug-induced liver injury (iDILI), human leucocyte antigens, HLA, iDILI genetics, idiosyncratic hepatocellular drug-induced liver injury, flucloxacillin, cytochrome P450, metabolic hypothesis, HLA B*57:01, immune-mediated hypothesis

## Abstract

Idiosyncratic drug-induced liver injury (iDILI) by flucloxacillin presents as both cholestatic and hepatocellular injury. Its mechanistic steps are explored in the present analysis as limited data exist on the cascade of events leading to iDILI in patients with an established diagnosis assessed for causality by the Roussel Uclaf Causality Assessment Method (RUCAM). Studies with human liver microsomes showed that flucloxacillin is a substrate of cytochrome P450 (CYP) with ist preferred isoforms CYP 3A4/3A7 that toxified flucloxacillin toward 5′-hydroxymethylflucloxacillin, which was cytotoxic to human biliary epithelial cell cultures, simulating human cholestatic injury. This provided evidence for a restricted role of the metabolic CYP-dependent hypothesis. In contrast, 5′-hydroxymethylflucloxacillin generated metabolically via CYP 3A4/3A7 was not cytotoxic to human hepatocytes due to missing genetic host features and a lack of non-parenchymal cells, including immune cells, which commonly surround the hepatocytes in the intact liver in abundance. This indicated a mechanistic gap regarding the clinical hepatocellular iDILI, now closed by additional studies and clinical evidence based on HLA B*57:01-positive patients with iDILI by flucloxacillin and a verified diagnosis by the RUCAM. Naïve T-cells from volunteers expressing HLA B*57:01 activated by flucloxacillin when the drug antigen was presented by dendritic cells provided the immunological basis for hepatocellular iDILI caused by flucloxacillin. HLA B*57:01-restricted activation of drug-specific T-cells caused covalent binding of flucloxacillin to albumin acting as a hapten. Following drug stimulation, T-cell clones expressing CCR4 and CCR9 migrated toward CCL17 and CCL25 and secreted interferon-γ and cytokines. In conclusion, cholestatic injury can be explained metabolically, while hepatocellular injury requires both metabolic and immune activation.

## 1. Introduction

Idiosyncratic drug-induced liver injury (iDILI) by multiple drugs and drug classes is a multifaceted disease that occurs in susceptible individuals after use of conventional drugs [1,2] and requires careful case evaluation to reduce the variables that commonly confound iDILI [3,4,5,6,7,8,9,10,11,12]. It is a typical human disease not reproducible in animal models that cannot mimic human genetics. The complexity of iDILI has attracted the interest of physicians and scientists with respect to mechanistic steps [13,14,15,16,17,18] and human genetic variability [19,20,21], including human leucocyte antigen (HLA) in patients with iDILI [21,22]; it is well evaluated for causality assessment using the Roussel Uclaf Causality Assessment Method (RUCAM) [6] in its original version from 1993 [23,24] or, better yet, its updated version from 2016 [25].

Drugs are in common use to treat or prevent diseases, and their efficacy by far outperforms adverse drug reactions (ADRs), including iDILI, which is defined as increased serum activities of alanine aminotransferase (ALT) above five times the upper limit of normal (ULN) or alkaline phosphatase (ALP) above two times the ULN, with diagnosis best established using the updated RUCAM [25].

Epidemiology issues of iDILI have been considered with respect to prevalence and incidence in previous publications [26,27,28,29,30,31,32]. Among these, special attention has been paid to well-conducted incidence studies of cohorts comprising iDILI patients [32,33,34,35,36]. With the exemption of one study [36], all others used cases assessed for causality [33,34,35] with either the traditional RUCAM [23,24] or the updated RUCAM [25]. When incidences were calculated per 100,000 individuals, the results for selected countries were as follows: France (13.9) [33], Nigeria (18.2) [34], Sweden (19.1) [35], and the United Kingdom (2.4) [36]. In these reports, antibiotics and non-steroidal anti-inflammatory drugs were the compounds most implicated [32]. With respect to flucloxacillin, the drug of special interest in this article, its iDILI incidence was estimated to be around 0.01% in one study only, without differentiation regarding evaluated populations [22]. For future assessment of epidemiology data in iDILI, the use of the updated RUCAM has been recommended [1].

Flucloxacillin causes rare RUCAM-based iDILI, with the following characteristics for 197 patients: gender (F/M) was 133/64, mean age 62 ± 13 (SD) years, mean time to onset 24 ± 18 days, and total days on drug 10 ± 6 days [22]. In this cohort, the pattern of liver injury was hepatocellular in 20%, mixed in 43%, and cholestatic in 38% of cases, while the RUCAM scoring was probable or higher in most cases.

The clinical outcome of iDILI is good, with complete resolution following instant cessation of the culprit drug, but in rare cases, acute liver failure, the need for liver transplantation, and vanishing bile duct syndrome may develop [3]. The US Acute Liver Failure Study Group determined that 12% of acute failure cases were caused by iDILI, but this percentage requires caution, as cases were not assessed by the RUCAM, and the study cohort lacked homogeneity through inclusion of non-drugs like herbal products [2,37].

In this analysis, the focus is on mechanistic steps leading to liver injury caused by flucloxacillin, an isoxazolyl penicillin antibiotic drug used in the Western world. For this approach, patients with iDILI by flucloxacillin were selected, for whom the diagnosis was confirmed by the use of the RUCAM. The aim was to close the mechanistic gap between the metabolic process via CYP 3A4/3A7 and the immune system in HLA B*57:01 genotype-positive patients with RUCAM-based hepatocellular iDILI.

## 2. Search Terms and Strategy

The literature search strategy included the PubMed database and Google Science, using the following terms: flucloxacillin AND idiosyncratic drug induced liver injury; flucloxacillin AND hepatocellular injury; flucloxacillin AND cholestatic liver injury; flucloxacillin AND Roussel Uclaf Causality Assessment Method. The first 50 publications derived from each term group were checked for their suitability to be included in this review article and provided the primary base for further analysis. The search was finalized on 31 August 2024. Limited to publications in the English language, there were no other restrictions regarding year of publication or study design.

Inclusion criteria of the review were primarily evidence-based cases of HLA B*57:01-positive patients with iDILI by flucloxacillin, and diagnosis was verified by causality assessment using the RUCAM. Secondarily, focus was extended to studies presenting data related to pathogenesis of iDILI by flucloxacillin. Excluded from analysis were clinical reports of iDILI by flucloxacillin evaluated by causality-assessing methods lacking scientific standards or internal and external validation.

## 3. Flucloxacillin among the Top Drugs Causing iDILI

Among the top drugs implicated in suspected iDILI is flucloxacillin, ranking with 130 cases at place 2 after amoxicillin-clavulanate with 333 cases [38]. These figures were derived from worldwide iDILI case reports published until 2018, all assessed for causality by the RUCAM. Most flucloxacillin cases came from Sweden (n = 129) [39] with one case from Germany [40], and ranked in Sweden nationwide at place 1 [39]. In 2019, 197 additional RUCAM-based cases of iDILI by flucloxacillin were reported; studied patients came from the UK (n = 159), Sweden (n = 37), the Netherlands (n = 3), and Australia (n = 1) [22].

## 4. Listing of iDILI by Flucloxacillin with Evidence Based on RUCAM and HLA

It is a privilege to have in our hands case data of 255 patients with iDILI by flucloxacillin; all were assessed for causality by the RUCAM. Thus, there is overwhelming support that flucloxacillin may cause liver injury, as evidenced by firm iDILI diagnosis (Table 1) [22,41,42,43].

The obvious strict focus on the RUCAM for assessing causality of iDILI cases may not be appreciated by all DILI experts in the field. In the past, causality methods others than the RUCAM were also applied in iDILI cases, and all of these had problems of internal and external validation or were not conceptualized for liver injury characteristics specified for drugs, as already thoroughly discussed in 2016 [25]. In more detail, both the Naranjo method and the WHO method are not qualified for evaluating liver injury by drugs as they are destined to assess general ADRs that may or may not include liver injury cases. The DILIN method fails to provide a scoring system for elements specific to iDILI and is based on subjective arbitrary opinions, known as a global introspective approach, in a similar way to the WHO method, conditions that prevent the required method validation [21,25], while the RUCAM received both internal and external validation and has advantages over the DILIN method [21].

## 5. Clinical Features of iDILI Due to Flucloxacillin with Evidence Based on RUCAM and HLA

All 255 cases were assessed for causality using the RUCAM and screened for association with HLA genes, which revealed that all patients showed a genetic association with HLA (Table 1), most with HLA B*57:01 [5,15,16,17] and few with HLA B*57:03 [22]. Consequently, the combination of the RUCAM with HLA alleles provided homogenous study cohorts [22,41,42,43] ready for further analysis that included clinical characteristics of iDILI by flucloxacillin. However, among the four homogeneous cohorts [22,41,42,43], only a single report described characteristics of the iDILI [22], in support of another report with iDILI cases assessed by the RUCAM but not for HLA association [44], with data of clinical features as compiled in Table 2 [22,44].

The study cohort listed in Table 1 is homogenous as all patients were examined in the context of the RUCAM [22,41,42,43], which asks for thresholds of ALT and ALP as well [23,24,25], among others. As an example, the threshold for ALT was 646 U/l (normal 4–43) and that of AST 349 (U/l (4–43) [43]. Liver histology is not part of the RUCAM algorithm but was provided in one study [43] out of four reports [22,41,42,43]. As assessed during hospitalization, there was slight multifocal, microvesicular steatosis, sinusoidal dilatation, marked congestion, hemorrhage, a multifocal collapse of hepatocytes, and an inflammatory infiltrate of the mononuclear type [43], likely to be classified as a T-cell, as shown by other peripheral blood studies [44]. Additional clinical features are given (Table 2) [22,44].

There was the challenging notion that, in patients with iDILI due to flucloxacillin, liver injury occurred up to two months after treatment was stopped [45]. This is an unusual situation that cannot be explained by the proposed mechanism of liver toxicity alone but may be due to immune cells in iDILI. Alternatively, these few patients may not have received a causality assessment using a method, such as the RUCAM, robust enough to firmly exclude alternative causes, such as comedicated herbs including herbal supplements or coexisting and undetected virus infections.

Excluding alternative causes in cases of suspected iDILI is a hallmark in assessing these liver injury cases; early observations from the UK [46,47] showed that up to 47% of initially diagnosed iDILI cases were not due to drugs but rather triggered by alternative causes unrelated to drug treatment [47].

## 6. Flucloxacillin as Substrate of Cytochrome P450

Flucloxacillin is a substrate of and metabolized by the hepatic microsomal cytochrome P450 (CYP) [38], primarily the CYP 3A4 and CYP 3A7 isoforms [48,49], while the other CYP 2C9 isoform fails to significantly contribute to the microsomal metabolism of flucloxacillin [50]. Similarly, other drugs are substrates of CYP, usually the CYP 3A4 isoform, responsible for their metabolism [38,48,49,50,51,52] and for the potential of metabolic interaction with flucloxacillin that may modify liver injury caused by flucloxacillin.

In at least 28/48 drugs (58.3%) causing RUCAM-based iDILI, clinical and experimental evidence showed that metabolism proceeds via hepatic microsomal cytochrome (CYP), whereas for the remaining 20 drugs (41.7%), there were negative or missing results implicating CYP in the metabolism of these drugs [28,48,49]. Drugs implicated in iDILI assessed for causality by the RUCAM were metabolized by the following CYP isoforms: 49.6% by CYP 3A4/5, 24.6% by CYP 2C9, 13.2% by CYP 2E1, 7.3% by CYP 2C19, 3.5% by CYP 1A2, and 1.8% by CYP 2D6 [38,48,49]. The primary source of the microsomal fraction is the endoplasmic reticulum of the hepatocyte, visible under electron microscopy [53]. The microsomal fraction is the biochemical counterpart of the endoplasmic reticulum, obtained through ultracentrifugation of the liver homogenate. It allows for the metabolism of most drugs and other products like ethanol [48].

The metabolism of drugs like flucloxacillin proceeds via the microsomal drug-metabolizing enzyme system located in the membranes of the smooth endoplasmic reticulum of the hepatocyte [51,52,53]. The system requires reduced nicotinamide-adenine-dinucleotide-phosphate (NADPH), oxygen, and as obligatory membrane components, cytochrome P450 (CYP) and NADPH-cytochrome P450 reductase, both representing protein components, as well as phospholipids in the form of phosphatidylcholine as a non-protein membrane component [54]. The phospholipid facilitates the transfer of electrons from NADPH-cytochrome P450 reductase to cytochrome P450, but it is not an electron carrier itself. Accordingly, the drug flucloxacillin is converted to its toxic metabolite 5′-hydroxymethylflucloxacillin, which participates in the development of liver injury by flucloxacilline [49].

Cytochrome P450 provides the binding site for molecular oxygen and the substrate, while the reductase functions as an electron carrier shuttling electrons from NADPH to cytochrome P450. Drugs acting as substrates like flucloxacillin undergo several steps during the catalytic cycle of cytochrome P450, leading to the generation of the oxidized substrate. Cytochrome P450 catalyzes the oxidation of substrates such as drugs like flucloxacillin, which binds to the ferric (3^+^) iron of cytochrome P450 as the initial metabolic step finally leading to the oxidized substrate. Shortly after the introduction of the second electron, an intermediate complex with radicals is generated that is essential for the toxification of substrates like flucloxacillin to 5‘-hydroxymethylflucloxacillin [38].

## 7. Role of CYP 3A4 in Converting Flucloxacillin for Toxic Metabolite Formation

Experimental evidence suggested that metabolite formation from flucloxacillin via CYP 3A4 rather than the parent chemical itself participates in iDILI, but CYP 3A7 was not evaluated under these specific experimental conditions [50,55]. Following microsomal metabolism of flucloxacillin, 5′-hydroxymethylflucloxacillin is found in the supernatant of the reaction medium, obtained through centrifugation. The reaction medium contained an NADPH (nicotinamide adenine dinucleotide phosphate)-generating system, human liver microsomes or recombinant human CYP 3A4, preincubated with flucloxacillin in the presence of molecular oxygen, and 5′-hydroxymethylflucloxacillin was identified as one major metabolite [55]. This can be produced in large amounts because CYP 3A4 is the most abundant CYP isoform in the liver, located in the hepatocytes [53] as opposed to human biliary cells [55]. 

The pharmacokinetics and oral bioavailability of flucloxacillin were studied in elderly hospitalized patients [56]. A single dose of intravenous or oral flucloxacillin sodium (500 mg) was administered in a random order on different occasions separated by at least 2 days. Blood and urine samples were taken up to 24 h after drug administration and levels of flucloxacillin and 5-hydroxymethylflucloxacillin as the major metabolite were measured by high-performance liquid chromatography. Flucloxacillin elimination, but not oral absorption, was reduced in the elderly, compared with data from young healthy subjects. Total clearance, renal clearance, and volume of distribution were 0.083 ± 0.013 L/kg/h, 0.038 ± 0.01 L/kg/h, and 0.184 ± 0.034 L/kg, respectively. Regression of flucloxacillin renal clearance (CL_cr_) on estimated creatinine clearance (CL_cr_) gave the following relationship: Cl_r_ = 0.755 (CL_cr_) + 10.6 (r = 0.91; *p* = 0.004). After intravenous administration, terminal half-lives for flucloxacillin and 5-hydroxymethylflucloxacillin were 2.21 ± 0.51 h and 3.0 ± 0.75 h, respectively. Flucloxacillin was absorbed rapidly after oral administration, with a mean absorption time of 0.95 ± 0.34 h and a time to reach peak concentration of 1.20 ± 0.29 h. The absolute bioavailability of flucloxacillin from capsules was 54.4 ± 18.8% [56].

## 8. Flucloxacillin-Modified Hepatocellular Proteins and Hapten Generation

**Figure 1:**
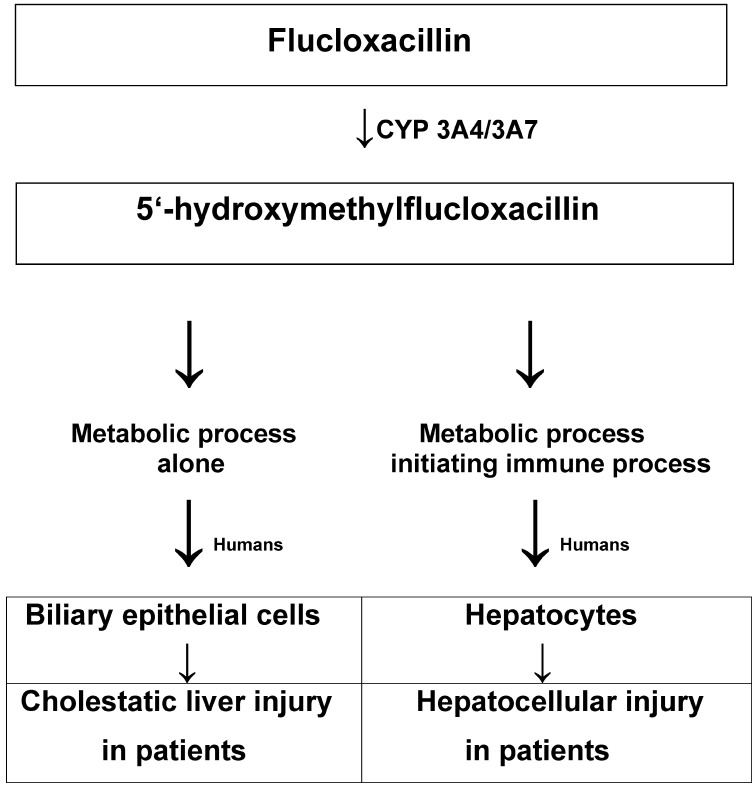
Metabolic role in initiating liver injury caused by the toxic intermediate of flucloxacillin. This proposal refers to patients with RUCAM-based iDILI after treatment with the antibiotic drug flucloxacillin. Illustrated is the dualistic process finally leading to liver injury: while cholestatic liver injury develops without participation of the hepatic immune system, hepatocellular injury is triggered by HLA B*57:01 and the hepatic immune system. Abbreviation: CYP, cytochrome P450.

In line with other drugs causing liver injury and looking at it on the molecular level [38,57], the NADPH-dependent conversion of flucloxacillin to 5′-hydroxymethylflucloxacillin, proceeding in the hepatic endoplasmic reticulum corresponding to the microsomal fraction, is associated with the generation of reactive oxygen species responsible for injury of microsomal and mitochondrial biomembranes [57,58,59]. Within these membranes, polyunsaturated fatty acids (PUFAs) are modified into lipid peroxides, thereby impairing the function of the subcellular organelles by processes termed mitochondrial and microsomal oxidative stress [38,58]. The various oxidative stress forms are the initial step leading to hepatocellular DILI [57,58,59], as shown by increased LTs in case reports [22,41,42,43]. The generation of ROS and the toxic metabolite of flucloxacillin is clearly shown in patients with DILI by flucloxacillin, in whom haptens are detected [60,61,62,63,64,65], in line with the concept of hapten-drug-specific T-cell activation [66], applicable to idiosyncratic DILI [14]. In addition, a non-hapten mechanism may exist because T-cell clones from healthy donors fail to interact via the hapten [65]. Based on experimental and clinical data, a model of mechanistic steps leading from metabolism of flucloxacillin to liver injury due to the use of flucloxacillin is presented (Figure 1) [14,55,59,60,61,62,63,64,65,66].

## 9. Immune-Based Cascade of Events Leading to Hepatocellular DILI by Flucloxacillin

The cascade of events leading to the immune-dependent idiosyncratic hepatocellular DILI caused by flucloxacillin use starts with the CYP 3A4/3A7-dependent metabolic generation of 5′-hydroxymethylflucloxacillin from the parent drug flucloxacillin (Figure 1) [50,55], characterized as a metabolic hypothesis [13,14,55,67]. During this NADPH-dependent metabolic process, radicals will be formed, involving the parent drug and its metabolite, which together with ROS commonly lead to a first injurious hit presenting as sterile inflammation due to the participation of pathogen-associated molecular patterns (PAMPs) that release damage-associated molecular patterns (DAMPs) [13,14]. Concomitantly, radicals formed during the drug-metabolizing process will irreversibly bind to the protein in the sense of haptenation [13,14,60,61,62,63,64,65,66], thereby acting as neo-antigens [14]. The immune response against the hapten is T-cell-dependent, a process that requires the uptake, processing, and presentation of peptides on major histocompatibility complex (MHC) class I molecules by antigen-presenting cells to the specific T-cell [67]. The HLA B*57:01 gene as an MHC class I molecule found in patients with hepatocellular DILI by flucloxacillin (Table 2) [22,44] is the perfect gene involved in this specific liver injury [64]. Immune processes in DILI due to flucloxacillin are based on the hepatic adaptive immune system, which requires HLA B*57:01, T-cell receptors, and prior transformation from the innate immune system, which in turn is activated by reactive metabolites and DAMPs [14]. These theoretical considerations of the relationship between iDILI and the immune system are substantiated by clinical evidence based on HLA B*57:01-positive patients with iDILI by flucloxacillin and verified diagnosis by the RUCAM, which provided the immunological basis for iDILI by flucloxacillin, although serological data on autoimmune parameters including anti-CYP 3A4 antibodies or anti-flucloxacillin antibodies were not provided [42]. As a result, an immunological role in liver injury was shown by HLA B*57:01-restricted activation of drug-specific T-cells. In more detail, naïve T-cells from volunteers expressing HLA B*57:01 were activated by flucloxacillin when the drug antigen was presented by dendritic cells [42]. In addition, following drug stimulation with flucloxacillin, T-cell clones expressing CCR4 and CCR9 migrated toward CCL17 and CCL25 and secreted interferon-γ (IFN-c), while T helper (Th)1 and 2 released cytokines and the cytolytic perforin, granzyme B, and FasL [43]. Flucloxacillin bound covalently to selective lysine residues on albumin, while the level of binding correlated directly with the stimulation of T-cell clones, and activation of CD81 clones with flucloxacillin was processing-dependent and restricted to HLA B*57:01 [50]. These data further elucidated the mechanistic cascade of events in HLA B*57:01-positive patients with RUCAM-based idiosyncratic hepatocellular DILI and closed the gap, starting with the metabolic generation of 5′-hydroxymethylflucloxacillin via the microsomal CYP 3A4 isoform followed by that of drug-specific T-cells by restricted HLA B*57:01. Thus, for hepatocellular DILI, and following the initial metabolic event, the T-cell-dependent immune mechanism, along with the activation of the innate immune system to the adaptive immune system, allows for the final step leading to hepatocellular injury (Figure 1). There is uncertainty regarding whether, in addition to metabolic pathways, as depicted (Figure 1), the parent drug may form the haptens [58].

Recently, mouse models with restricted HLA-B*57:01 presentation for the study of flucloxacillin-driven T-cell activation and tolerance in liver injury have been developed [68]. Using these in vivo models, flucloxacillin primes CD8^+^ T-cells to recognize drugs presented by HLA-B*57:01. Most importantly, the HLA-B*57:01-dependent CD8^+^ T-cell reaction to flucloxacillin is dependent on the presence of CD4^+^ cells, in the sense of regulatory T-cells. As a result, these experimental data additionally provide strong evidence that the clinically observed idiosyncratic hepatocellular DILI triggered by flucloxacillin proceeds via immune mechanisms under the participation of the HLA B*57:01 genotype found in patients with this special type of DILI.

## 10. Risk of iDILI Due to Flucloxacillin

Studies showed that the risk of flucloxacillin-induced liver injury increases in subjects with the HLA-B*5701 allele [22,44]. Based on a prevalence of 8.5 per 100,000 individuals, the positive predictive value of the HLA B*5701 allele test for liver injury is low [22,44], and predictive genetic testing for the reaction would be unfeasible, as 13,513 people would need screening to prevent one case [44]. Therefore, routine screening for this allele is not recommended in patients before initiation of a therapy with flucloxacillin. The heterogeneity in patient responses to the liver injury may reflect the involvement of other HLAs or non-genetic factors including environmental ones.

## 11. Biliary Injury in Experimental Studies and Cholestatic Liver Injury in Humans with iDILI

The metabolite 5′-hydroxymethylflucloxacillin was toxic to primary cultures of human gallbladder-derived biliary epithelial cells, as evidenced by lactate dehydrogenase release, signifying experimental biliary injury [50,55], and substantiated by its high biliary concentrations based on data from perfused rat livers [69]. In addition, flucloxacillin is a substrate for multidrug resistance-associated protein (MRP2), which concentrates the drug in the bile epithelia [14,70]. These experimental data are in line with those from patients who were treated with flucloxacillin and experienced iDILI, with a pure cholestatic liver injury pattern observed in 39% of a large RUCAM-based iDILI cohort of 197 patients [22] and confirmed in another RUCAM-based study with 83% of patients ≥70 years vs. 56% of patients <70 years old [44]; additional information is shown above (Table 2). The higher susceptibility to cholestatic liver injury in older age may be caused by comedication, changes in hepatic blood flow, or diminished liver function capacity. The evolution of cholestatic liver injury can be traced back to biliary injury, classifying experimental liver injury as a typical metabolic result, considering that flucloxacillin is a substrate of and metabolically toxified by CYP 3A4/3A7 (Figure 1).

## 12. Strength and Limitation of the Review

The strength of this report is in its focus on immune mechanisms triggered by metabolic CYP 3A4/3A7 involved in idiosyncratic hepatocellular DILI in patients with HLA B*57:01 with a diagnosis confirmed by the RUCAM. However, a limitation exists because the proposed mechanistic steps may not fully mirror the pathophysiology due to the fact that 17% of iDILIs by flucloxacillin are observed in patients who lack HLA B*57:01 gene mutation, requiring additional studies.

## 13. Conclusions

There is now convincing experimental and clinical evidence that the idiosyncratic cholestatic liver injury and hepatocellular injury caused after treatment with the antibiotic flucloxacillin follow two different cascades of events. Cholestatic liver injury is the result of the metabolic toxification of flucloxacillin to 5′-hydroxymethylflucloxacillin via CYP 3A4/3A7 in the absence of hepatic immune cells. With respect to hepatocellular injury, flucloxacillin or its metabolite functions as an antigen and causes covalent binding to proteins, providing clinical evidence that the first step of the injury is triggered metabolically through the CYP 3A4/3A7 isoform, and the second step involves immune cells secreting cytokines and interferons that allow for the activation of the innate immune system to the adaptive immune system. The elucidation of these dualistic steps was clinically achieved in HLA B*57:01-positive patients with idiosyncratic DILI by flucloxacillin treatment and verified diagnosis by the RUCAM, and additional studies are proposed to help verify the proposed mechanistic steps involved in liver injury by flucloxacillin.

## Figures and Tables

**Table 1:** Flucloxacillin causing iDILI assessed for HLA association and causality by RUCAM.

DRUG	HLA Allele	RUCAM-Based iDILI Cases (n)	RUCAM-Based Causality	First Author
Flucloxacillin	B*57:01	51	4/51 patients had a possible causality, 18 a probable causality, and 29 a highly probable causality grading	Daly, 2009 [41]
Flucloxacillin	B*57:01	6	2/6 patients had a possible causality, 2 a probable, and 2 a highly probable causality	Monshi, 2013 [42]
Flucloxacillin	B*57:01 B*57:03	197	22/197 patients had a possible causality, 90 a probable, and 85 a highly probable causality grading	Nicoletti, 2019 [22]
Flucloxacillin	B*57:01	1	Score 8, probable causality	Teixera, 2020 [43]

Abbreviations: iDILI, idiosyncratic drug-induced liver injury; HLA, human leucocyte antigen; RUCAM, Roussel Uclaf Causality Assessment Method.

**Table 2:** Clinical data of patients with RUCAM-based iDILI caused by flucloxacillin.

Clinical Specifics of iDILI by Flucloxacillin Based on RUCAM and HLA B*57:01 [22]	Clinical Specifics of iDILI by Flucloxacillin Based on RUCAM [44]
General: Derived from the original cohort of 197 patients; only those with firmly established HLA B*57 positivity (=163; 83%) were considered, excluding patients with confirmed HLA B*57 negativity (n = 34; 17%).	General: The study cohort was derived from the UK Clinical Practice Research Datalink. Patients were assessed by the RUCAM but not for association with HLA alleles.
With 114/163 cases, the female gender prevailed (70%).	With 125/861.962 cases, the female gender prevailed (57%).
Age at onset was 63 ± 13 years (mean ± SD); time to onset 23 ± 13 days (mean ± SD); total days on drug 10 ± 5 days (means ± SD).	Age at onset was 48 years (34–65) (median IQR); time to onset 23 ± 13 days (mean ± SD); total days on drug 10 ± 5 days (means ± SD).
Liver injury pattern: hepatocellular, 31/163 cases (19%); cholestatic, 63/163 (39%); mixed, 69/163 (42%).	Liver injury pattern: cholestatic in 38/46 (83%) of patients ≥70 years, and in 15/27 (56%) of patients <70 years.
RUCAM-based scoring: Highly probable, score ≥8 (72/163 cases; 44%); probable, 6–8 (73/163; 45%); possible, 3–5 (18/163; 11%).	RUCAM-based scoring: probable, score 6–8 (63/73; 86%); possible, score 3–5 (10/73; 14%).
Risk of iDILI: An increased risk was shown for ages >70 years, female gender, and HLA B*57:01.	Risk of iDILI: An increased risk was observed with ages >70 years, repetitive use of flucloxacillin, and co-medication.
Prevalence: NA	Prevalence: Based on a prevalence of 8.5 per 100,000 individuals, predictive genetic testing for the reaction would be unfeasible, as 13,513 people would need screening to prevent 1 case.

Data were extracted from a previous open access report [5]. For the listed parameters, there were no statistically significant differences between the cohort of HLA B*57:01-positive and the one of HLA B*57:01-negative patients. Abbreviations: NA, not available; RUCAM, Roussel Uclaf Causality Assessment Method.
